# The role of modelling and analytics in South African COVID-19 planning and budgeting

**DOI:** 10.1371/journal.pgph.0001063

**Published:** 2023-07-03

**Authors:** Gesine Meyer-Rath, Rachel A. Hounsell, Juliet RC Pulliam, Lise Jamieson, Brooke E. Nichols, Harry Moultrie, Sheetal P. Silal

**Affiliations:** 1 Faculty of Health Sciences, Health Economics and Epidemiology Research Office, University of the Witwatersrand, Johannesburg, South Africa; 2 Department of Global Health, School of Public Health, Boston University, Boston, Massachusetts, United States of America; 3 South African DSI-NRF Centre of Excellence in Epidemiological Modelling and Analysis (SACEMA), Stellenbosch University, Stellenbosch, South Africa; 4 Department of Statistical Sciences, Modelling and Simulation Hub, Africa, University of Cape Town, Cape Town, South Africa; 5 Nuffield Department of Medicine, Centre for Tropical Medicine and Global Health, University of Oxford, Oxford, United Kingdom; 6 Department of Medical Microbiology, Amsterdam University Medical Center, Amsterdam, the Netherlands; 7 Foundation for Innovative New Diagnostics, Geneva, Switzerland; 8 National Institute for Communicable Diseases (NICD), a division of the National Health Laboratory Service, Johannesburg, South Africa; Soongsil University, KOREA, REPUBLIC OF

## Abstract

**Background:**

The South African COVID-19 Modelling Consortium (SACMC) was established in late March 2020 to support planning and budgeting for COVID-19 related healthcare in South Africa. We developed several tools in response to the needs of decision makers in the different stages of the epidemic, allowing the South African government to plan several months ahead.

**Methods:**

Our tools included epidemic projection models, several cost and budget impact models, and online dashboards to help government and the public visualise our projections, track case development and forecast hospital admissions. Information on new variants, including Delta and Omicron, were incorporated in real time to allow the shifting of scarce resources when necessary.

**Results:**

Given the rapidly changing nature of the outbreak globally and in South Africa, the model projections were updated regularly. The updates reflected 1) the changing policy priorities over the course of the epidemic; 2) the availability of new data from South African data systems; and 3) the evolving response to COVID-19 in South Africa, such as changes in lockdown levels and ensuing mobility and contact rates, testing and contact tracing strategies and hospitalisation criteria. Insights into population behaviour required updates by incorporating notions of behavioural heterogeneity and behavioural responses to observed changes in mortality. We incorporated these aspects into developing scenarios for the third wave and developed additional methodology that allowed us to forecast required inpatient capacity. Finally, real-time analyses of the most important characteristics of the Omicron variant first identified in South Africa in November 2021 allowed us to advise policymakers early in the fourth wave that a relatively lower admission rate was likely.

**Conclusion:**

The SACMC’s models, developed rapidly in an emergency setting and regularly updated with local data, supported national and provincial government to plan several months ahead, expand hospital capacity when needed, allocate budgets and procure additional resources where possible. Across four waves of COVID-19 cases, the SACMC continued to serve the planning needs of the government, tracking waves and supporting the national vaccine rollout.

## Introduction

As of May 2022, South Africa has experienced four waves of COVID-19, with an official tally of more than 3,711,000 cases and 101,000 reported deaths [1]. The country reported its first imported COVID-19 case on 5 March 2020, with subsequent rapid spread into all districts in the country. In response, the South African government implemented a five-level COVID-19 alert system, beginning with a full lockdown (Level 5) from late March 2020 [2]. The alert levels determined the extent of restrictions to be applied during the national state of disaster, which was initiated in March 2020 and ended two years later, in April 2022 [2]. The risk-adjusted approach was guided by several criteria, including numbers of infections and rate of transmission, health facility capacity, the extent of the implementation of public health and social measures (PHSM), as well as the economic and social impact of continued restrictions [2].

The South African COVID-19 Modelling Consortium (SACMC) was established at the end of March 2020 in response to a request by the South African National Department of Health (NDOH) to project the spread of the disease to support policy and planning in South Africa over the course of the epidemic. The consortium developed two models to project incidence, deaths, need for hospital beds at different levels of care and the corresponding resources required. The models incorporated available data on COVID-19 cases, severity and mortality, and served to inform the public and a range of decision makers, including the Ministerial Advisory Committee on COVID-19 (MAC) advising the Minister of Health, staff in the NDOH and National Treasury, officials in the provincial departments of health and the private health sector.

This paper describes the 2-year process of continuously updating the models while collaborating with the diverse partners. The paper further provides an overview of the model results and the consortium’s recommendations at the different stages of the South African COVID-19 epidemic. The aim is to highlight the dynamic, multidisciplinary nature of policy-driven modelling of an emergency in a country with severely constrained resources. A full description of the models developed during wave 1 is provided in [3], and our analysis of COVID-19 related hospitalisations in [4].

## Methods

### The South African COVID-19 Modelling Consortium

The SACMC is a group of researchers from academic, non-profit and government institutions across South Africa. Established in late March 2020 by the NDOH, its mandate is to provide, assess and validate model projections to be used for planning purposes by the Government of South Africa. The SACMC’s work is coordinated by the South African National Institute for Communicable Diseases (NICD), which maintains the datasets used by the SACMC’s models. In addition to its core group of experienced infectious disease modellers and health economists, the SACMC convenes experts across a range of disciplines to provide insights, guide the selection of appropriate parameter values, ensure a close alignment to current clinical practice and sense-check model outputs.

### Policy-driven modelling

Since its establishment in March 2020, the SACMC has provided policy-driven modelling and analytics support in response to the evolving priorities of decision makers across the different stages of the epidemic. Several tools were developed and adapted over time to meet these needs. Taken together, these tools supported the South African government at national and provincial levels to conduct timely resource planning, shift scarce resources and implement appropriate PHSM.

At the start of the epidemic, the most pressing need was for short- and long-term projections of COVID-19 cases, including the number of severe and critical cases requiring hospital admission, and deaths under different PHSM scenarios. To fulfil these needs, the SACMC developed the National COVID-19 Epi Model (NCEM), a compartmental transmission model following a generalised Susceptible-Exposed-Infectious-Removed structure that accounts for disease severity (asymptomatic, mild, severe and critical cases) and treatment pathways (outpatient, inpatient non-ICU and ICU care) [3].

The National COVID-19 Cost Model (NCCM), a companion model, used epidemiological outputs from the NCEM on the number of mild, severe and critical cases. Based on this, it projected total COVID-19 resource needs and the associated impact on the national and provincial health budgets by incorporating information on the need for inpatient and outpatient resources (including their baseline availability and how to scale availability with the size of the epidemic). Resource projections covered drugs, diagnostics, ventilators, oxygen supply, field hospitals and other hospital infrastructure, staffing requirements and additional mortuary space. Model extensions included the quantification and cost of vaccines under different vaccination scenarios. These projections informed the development of resource quantifications and budgets, allowing timely negotiation with manufacturers and preparation of contracts for the additional resources anticipated based on precise quantifications. The exact volumes required could be regularly updated based on the latest model outputs.

**Fig 1:**
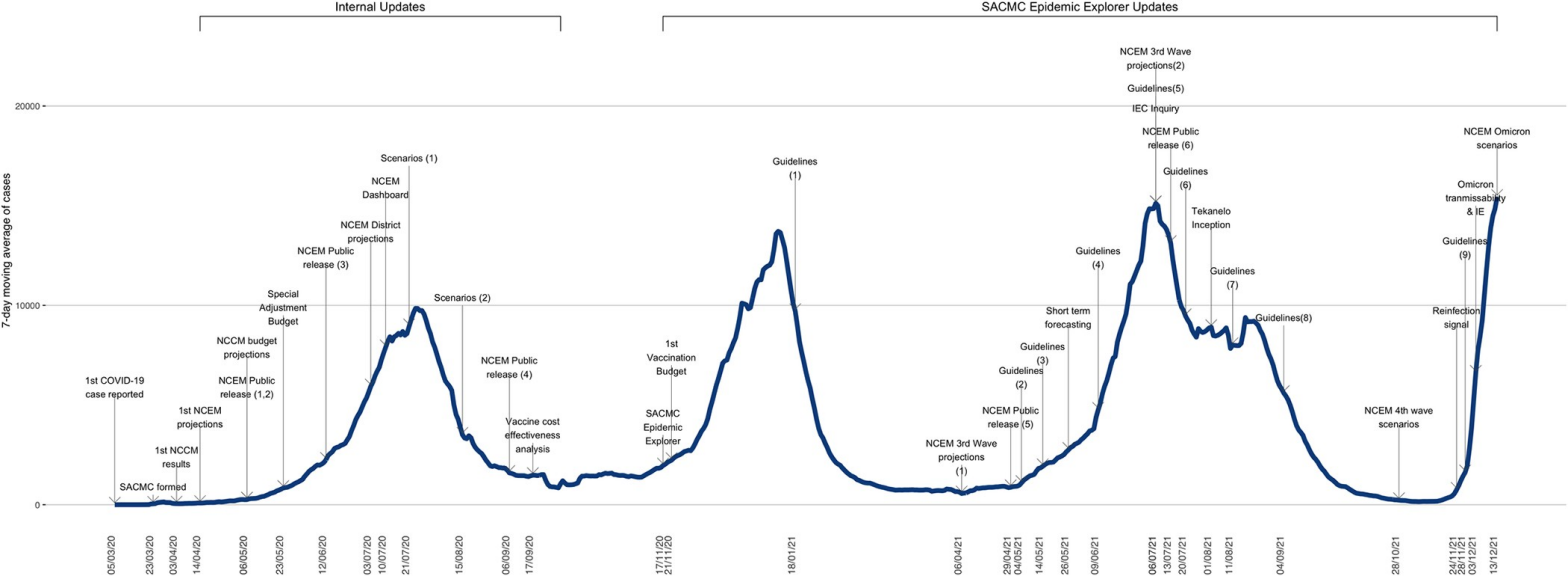
Select SACMC modelling and analysis contributions: Timeline from March 2020 to December 2021. See Table A in S1 Appendix for additional information.

As the epidemic progressed, in addition to the ongoing projections described above, priority was placed on resurgence monitoring, estimating the impact of relevant emerging variants of concern and modelling to inform the government’s vaccination procurement and rollout strategy. Between March 2020 and December 2022 in South Africa, apart from the wildtype virus that drove the first case wave, there were three dominant variants of concern: Beta, B.1.351, first detected in South Africa; Delta, B.1.617.2; and Omicron, B.1.1.529, which was again first identified in South Africa. Fig 1 shows the tools developed over the course of the epidemic in South Africa and the timing of their main outputs.

### Use of modelling

The SACMC’s modelling outputs have been used by a range of strategic and operational decision makers (Table 1). Different departments within the NDOH used NCEM outputs for purposes ranging from the quantification of drug volumes required for inpatient and outpatient care by the Affordable Medicines Directorate to the estimation of additional mortuary and graveyard spaces by the Environmental Health Directorate. Private sector initiatives such as the National Ventilator Project, which sourced ventilation equipment for public and private hospitals in South Africa, also used our model outputs. In addition, analysts coordinated by the Reserve Bank, the South African central bank, used SACMC model outputs to predict the macro-economic impact of the epidemic under different scenarios.

**Table 1:** Uses of NECM and NCCM outputs.

User	Purpose
NDOH:	
Ministerial Advisory Committee	Policy advice
Facility Readiness Committee	Number of beds and expansion of facilities
Human Resources for Health team	Staff required per level
National Ventilator Project	Number of ventilators needed
Oxygen planning team	Oxygen required
Affordable Medicines Directorate	Drug quantities
National Health Laboratory Service	Number of tests and testing priorities
Environmental Health Directorate	Number of mortuary containers
Provincial DOHs	Local planning and resource quantification
National Treasury	COVID-19 health budgets

The resurgence monitoring tool was used by planners and technical advisory teams to declare a resurgence of cases and respond according to the guidelines detailed in the NDOH Resurgence Plan [5]. The third and fourth wave scenario modelling and short-term forecasts were primarily used to estimate hospital admissions ahead of a third wave driven by the Delta variant in June 2021, and a fourth wave driven by the Omicron variant in November 2021.

Outputs of the NCCM and vaccination cost models were used by the National Treasury to make decisions regarding the additional budget allocation required for COVID-19. Model outputs, in part, informed a budget allocation of 1.42 billion USD for COVID-19 specific health care announced by the South African president at the end of April 2020 [6], most of which was financed through the reallocation of the existing health budget [7]. Additionally, a group of experienced public finance specialists was trained to work with provincial Departments of Health to update the model with provincially specific data. This included the baseline availability of resources, prices and need for resources that were independent of the course of the epidemic, such as personal protective equipment and isolation and quarantine facilities.

### Key parameters and data sources

The parameter values for the early versions of the NCEM were based on literature and data from other countries as well as local expert opinion regarding the types, duration and outcomes of hospital treatment. Parameter values and assumptions were regularly updated as the scientific knowledge base on COVID-19 expanded. As South African data became available, parameters were adjusted to reflect the local context. Parameter selection was guided by ongoing input from clinicians, virologists, intensive care specialists, immunologists and epidemiologists on the SACMC. Table 2 contrasts the initial set of parameter values from late April 2020 and the updated set from September 2020; Table 3 provides an overview of the main data sources.

**Table 2:** Key NCEM parameter values and their evolution between April and September 2020.

Parameter	April 2020		September 2020	
	Value* (range)	Sources	Value* (range)	Sources
Infection severity and transmission**				
Proportion of cases that are asymptomatic	75%	[9–11]	75% (70%–80%)	[8–11]
Relative infectiousness of asymptomatic cases	-	-	80% (77.5%, 82.5%)	[12–14] Estimated through calibration to admissions and fatalities count data (DATCOV) [15]
Mild to moderate cases among the symptomatic	(95.64%, 96.78%)	adjusted based on [17]	(94.55%–97.13%)	Estimated through calibration to admissions and fatalities count data (DATCOV) [15,16]
Severe cases among the symptomatic	(2.46%–3.64%)		(2.58%–5.00%)	
Critical cases among the symptomatic	(1.16%–1.45%)		(0.18%–0.55%)	
Proportion of cases that are fatal	(0.30%, 0.412%)	[17,18]		
**Timeframes & treatment duration**				
Time from infection to onset of infectiousness	4 days (2–9)	[18,19–23], with additional input from analysis of NICD data	2 days (1–3)	[24–34] with input from the National COVID-19 Modelling Consortium
Time from onset of infectiousness to onset of symptoms	2 days (1–4)		4 days (3–5)	
Duration of infectiousness from onset of symptoms	5 days		5 days (4–6)	[35,36]
Time from onset of mild symptoms to testing	4 days (2–4)		4 days (3–5)	[25,26,18–21,23]
Time from onset of symptoms to hospitalisation	5 days (4–8)		5 days (4–6)	
Time from onset of symptoms to ICU admission	9 days (8–17)		see below	
Duration of hospital stay	12 days (7–16)			
Duration from ICU admission to discharge	18 days (14–18)			
Duration from ICU admission to death	5 days (4–7)			
Time in non-ICU (never ICU) to death/recovery			8 days (4–12)	Lengths of stay: values and ranges sourced from NICD COVID-19 Hospital Sentinel Surveillance database (DATCOV) [15,4]
Time in non-ICU for those destined for ICU			0 days (0–2)	
Time in ICU for those ventilated and destined to die			14 days (7–27)	
Time in ICU for those never ventilated and destined to die			11 days (7–18)	
Time in ICU for those ventilated and recovered			19 days (15–37)	
Time in ICU for those never ventilated and recovered			5 days (1–10)	
Time in non-ICU for those who were in ICU and recovered			0 days (0–6)	

**Table 3:** Summary of NCEM data sources.

National case and hospitalisation data from the South African National Institute for Communicable Diseases
Statistics South Africa projected 2020 district population projections [37]
Coronavirus COVID-19 (2019-nCoV) Data Repository for South Africa, Data Science for Social Impact Research Group @ University of Pretoria [38]
Medical Research Council analyses of weekly excess deaths [39,40]
Vaccine uptake and coverage from the National Department of Health’s Electronic Vaccination Data System
Vodacom Mobile Event Database
Google COVID-19 Community Mobility Reports
Published and pre-print academic literature (cited in Table 2)
Expert input from members of the SA COVID-19 Modelling Consortium and press releases and notices from the South African government’s COVID-19 Online Resource and News Portal (https://sacoronavirus.co.za/category/press-releases-and-notices/)

The NCCM used three types of input data: 1) The type and required quantities of resources, such as the number of inpatient beds projected by the NCEM, human resources at all care levels, oxygen, oxygen delivery devices, SARS CoV-2 tests, infection control and prevention infrastructure; 2) the public-sector prices of these resources; and 3) the baseline volume of resources available for the COVID-19 health response for all items where the quantities required exceeded existing resource levels, such as in the case of hospital beds or ventilators. Costs were evaluated from the provider perspective, in this case the South African government. Despite the need to provide COVID-19 testing and care in both the public and the private sectors, this perspective is appropriate given that contracting arrangements were put in place to ensure private-sector services were offered at charges similar to public-sector prices. As a result, we used public-sector prices and salaries throughout, based on the most recent tariffs and public tenders (Table 4).

**Table 4:** Summary of NCCM data sources.

Intervention/ ingredient	Type of target population	Data sources		
		Target population	Cost	Baseline capacity
PPE	Healthcare workers at all levels	DHIS	Own analysis	NDOH
ICU beds, staff and linen	Critically ill cases	NCEM	Own analysis	NDOH
Ventilators	Critically ill cases (subset)	NCEM	Public tender	NDOH
Testing	All cases who present for testing	Own analysis	NHLS	NHLS
Community health workers (CHW)	10,000	DHIS	Public salaries	-
CHW supplies	Thermometers for 10,000 CHW	-	Public tender	-
Isolation		-	Own analysis	-
O_2_	Severely + critically ill cases	NCEM	Own analysis	NDOH
Hospital beds, staff and linen	Severely ill cases	NCEM	Own analysis	-
Drugs	All cases (by level of severity)	NCEM	Own analysis	-
PHC staff	70% of symptomatic mild cases	NCEM	Own analysis	-
Fever clinics	NDOH	NDOH	NDOH	-
30-bed wards	NDOH	NDOH	NDOH	-
Mortuary cupboards and cabinets	Deaths by municipality	NCEM	NDOH	NDOH

### Evolution of modelling purpose, structures and tools across the epidemic

#### Projections and scenarios for short- and long-term COVID-19 burden

Government set three priorities for modelling and projections during the first wave of the COVID-19 epidemic in South Africa: 1) to generate short- and long-term projections of COVID-19 cases, estimating the pace at which cases might increase and spread between provinces; 2) to project the expected number of severe and critical cases leading to hospital admission, as well as estimates of the corresponding resource requirements; and 3) to compute the cost of the health sector response to the epidemic at a provincial and national level in order to inform the adjustment of the health budget and the flow of resources around the country.

**Fig 2:**
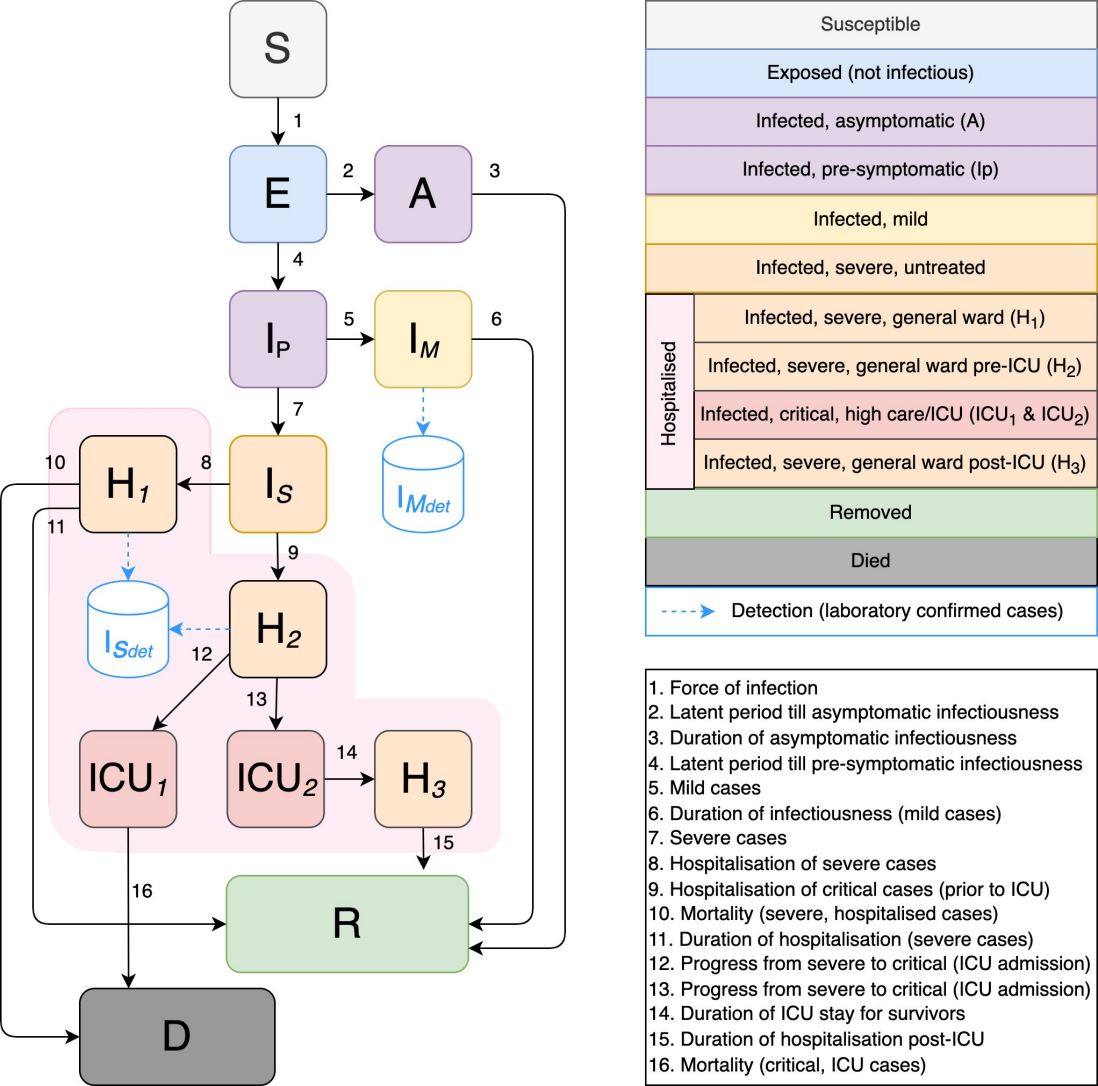
Original NCEM model structure (v1).

To fulfil the first and second priority, we developed the NCEM, a compartmental transmission model to estimate the total and reported incidence of COVID-19 in the nine provinces (and later, 52 districts) of South Africa. It was designed to simulate the impact of different behavioural scenarios, inform resource requirements and predict where gaps could arise based on the available resources within the South African health system. The model follows a generalised Susceptible-Exposed-Infectious-Removed (SEIR) structure accounting for disease severity (asymptomatic, mild, severe and critical cases) and the treatment pathway as shown in Fig 2.

Version 1 of the NCEM v1, stratified at the provincial level, accounted for the clinical profile of SARS-CoV-2 and the hospital care pathway (Figure A in S1 Appendix). With limitations on general ward and ICU capacity, the model was extended in v2 to account for limited access to hospital level care and restricted capacity in wards at the provincial level (Figure B in S1 Appendix). With a need for planning support at a finer spatial granularity and lifting of restrictions on intra-country travel, a stochastic version of the NCEM (v3) was extended to cover the 52 districts of South Africa, linked through a connectivity matrix formulated by mobile data over time. NCEM v4 was necessary after the detection of the Beta variant in South Africa (Figure C in S1 Appendix). The model was further stratified to include 7 age groups across three subpopulations of interest: healthcare workers, the population with comorbidities and everyone else. Vaccination was included to incorporate a generic vaccine’s potential effectiveness against infection and severity. In anticipation of another variant and with the vaccination programme underway for healthcare workers, NCEM v5 was developed to include a third variant stratification for this hypothetical new variant. The model was updated at the beginning of the third wave to represent early data on the characteristics of the Delta variant (Figure D in S1 Appendix). With NCEM v6, vaccination was included in greater detail, with specific vaccination types based on those planned to be locally administered and additional differentiation between vaccine effectiveness in those with prior infection vs the immunologically naïve (Figure E in S1 Appendix). Lastly, just the NCEM updated to v7 just before the fourth wave with a fourth stratification for another hypothetical new variant. This produced various scenarios driven by different joint assumptions regarding its transmissibility and immune escape properties (Figure F in S1 Appendix). This version was updated to the characteristics of the Omicron variant within days of Omicron being named. Model versions v4-7 were updated to represent vaccine coverage and uptake across provinces and age groups at each time point based on vaccination data from the National Department of Health.

The NCEM was originally developed as a deterministic compartmental model, where the characterisation of uncertainty changed over the course of the pandemic. Early on in the first wave, when uncertainty in parameter values was large and the least complex framework of the NCEM was implemented, uncertainty in parameter values was explored through sensitivity analyses and uncertainty bounds estimated through random draws from specified parameter distributions. When the need arose to expand the model to a finer spatial granularity in v3, the NCEM was additionally implemented stochastically to account for greater uncertainty at small geographical levels. The inclusion of variants and vaccines into NCEM versions 4 onwards brought an additional layer of uncertainty in projection modelling, and scenario analyses were employed to depict uncertainty in the outcomes of unknown vaccine and variant characteristics.

#### Resource planning

The National COVID-19 Cost Model (NCCM) takes inputs from the NCEM and cost inputs based on data from existing sources that were adapted to represent the type, number and prices of ingredients required in South Africa’s COVID-19 response. It calculates annual budgets for the NDOH’s response to COVID-19, allocating costs at the level of the provinces as well as NDOH, incremental to existing resources such as hospital beds and staff contingents.

The NCCM was updated with new NCEM results when they became available and was changed to incorporate additional interventions when deemed relevant by policy makers and planners (for example, temporary inpatient infrastructure such as field hospitals and add-on clinic space). Additional updates included new clinical interventions once they were incorporated into national COVID-19 management guidelines (such as dexamethasone treatment and high-flow nasal cannulae treatment), and prices and quantities as new tenders and data on actual resource use became available. For example, in July 2020 we adjusted our assumptions regarding inpatient length of stay downwards from the initial estimates (which were based on international literature) to results from our analysis the South African hospital sentinel surveillance database [15] (for more detail regarding the methods see [4]); and inpatient costs were updated based on a more detailed analysis of South African ingredients and prices [41].

#### Dashboard for disseminating model updates

Between early April and early September 2020, the NCEM was updated frequently and results made available to stakeholders within the South African government. Reports on a subset of these updates were additionally made public. To aid the timely dissemination of projections, we developed a web-based interactive application, the National COVID-19 Epi Model Dashboard (https://masha-app.shinyapps.io/NCEMDashboard), which visualised the most important NCEM outputs on projected cases, hospitalisations and deaths. These included active cases and cumulative detected cases for symptomatic, severe and critical cases; hospital beds needed and cumulative admissions for non-ICU, ICU ventilated and ICU non-ventilated; and cumulative deaths.

#### Resurgence monitoring

At the beginning of the second wave of COVID-19 cases in September 2020, driven by the Beta variant which originated in South Africa, resurgence monitoring became a priority. The SACMC assisted in the development of a set of resurgence metrics to support planners and technical advisory teams to declare a resurgence or wave and respond according to the guidelines detailed in the Ministry of Health’s resurgence action plan [5]. To facilitate effective communication and dissemination of these metrics, we developed a second web-based dashboard called the SACMC Epidemic Explorer in two versions: a sub-district level version to guide planners at all government levels, and a district-level version (www.SACMCEpidemicExplorer.co.za) to inform the public. It allows users to explore the COVID-19 epidemic in South Africa, analyses resurgence risk, monitor confirmed COVID-19 hospital admissions and deaths, and presents metrics to prepare for future outbreaks, including estimates of case levels, percentage change in cases and periods of consistent growth. We measured the metrics’ performance against three main criteria: whether the resurgence metrics were consistent in their messaging; whether the metrics provided sufficient early warning; and whether the metrics relaxed the severity of their messaging at a suitable time and pace after the peak of the epidemic.

#### Short-term forecasting

Towards the end of the second wave, short-term forecasts of COVID-19 cases, hospital admissions, and of the likelihood of when provinces would meet the third, then fourth wave criterion were developed. This enabled improved situational awareness and informed government resource planning. These two-week case and admission forecasts were updated weekly and three times weekly, respectively, and displayed on the SACMC Epidemic Explorer for easy access.

## Results

### Findings of the National COVID-19 Epi Model (First wave)

In May 2020, using version 1, the model projected an estimated 8.01 and 8.62 million laboratory-confirmed cases and 40,223 and 43,759 deaths in the optimistic and pessimistic scenarios, respectively, by 1 October. Active cases were estimated to peak in early July in the pessimistic, and mid-July in the optimistic scenario, with a maximum number of between 72,281 and 77,899 hospital beds and 31,656 and 24,150 ICU beds in use at peak. Total cumulative incidence was estimated to reach between 48.7 and 51.7 million cases (symptomatic or asymptomatic) by 1 October (i.e., an attack rate of 82–88%), with between 8.01 and 8.62 million detected cases, assuming a detection factor of 1 in 6 cases. While these projections incorporated a certain level of effectiveness of PHSM in their scenarios, they underestimated the severity and effect of government restrictions and population adherence to them and thus assumed that all exposure would happen during a single wave. A full set of projections for all nine provinces is available in our detailed reports [42,43].

The updates in version 2 took into account the variation in timing and level of peaks of the epidemics between the provinces and between the districts in each province. This resulted in an estimated national peak in cases at a a similar time (i.e., mid-August 2020) to the optimistic scenario from version 1, but at a lower level. While the model projected a concomitant lower peak in the need for hospital (non-ICU) and ICU beds at a national level, bed capacity was still expected to be breached or overwhelmed in all provinces. We noted that increasing capacity to accommodate patients in hospital could allow the country to better leverage new therapeutic options, such as high-flow oxygen and dexamethasone, which had the potential to improve mortality outcomes.

In version 3, we modelled the impact of four behavioural scenarios on the four provinces with the most advanced epidemics: Western Cape, Eastern Cape, Gauteng and KwaZulu-Natal. The application of each of these behavioural scenarios led to earlier and/or lower peaking of cases than our original projections. One scenario (in which the behavioural response threshold was assumed to be 110 deaths per day) peaked at roughly the same level but shifted the peak forward. The full analysis detailing the behavioural scenarios is available in [44].

The updated version 4 from early September 2020 estimated that there had been 15.2 million infections by September, equating to 25.5% (uncertainty range: 22.0%–28.6%) of the population—a much lower first-wave attack rate than estimated in our first version. Under the moderate testing scenario, cumulative detected cases were estimated to continue to grow until between 570,000 and 1.2 million cases by early November (and only marginally so thereafter), depending on testing rates. The peak number of general hospital (i.e., non-ICU) beds in use was estimated to have been reached in early-August, at around 8,000 beds (when we had projected that around 12,500 beds would have been needed). The peak number of ICU beds in use was estimated to have been reached around the same time, with around 1,100 beds—although more than 2,000 beds would have been needed. Total deaths are estimated to continue to increase until early November when the cumulative number of all deaths would reach 37,000 (of which 16,000 would have been in hospital); thereafter the growth rate was estimated to be very low [44].

### Findings of the National COVID-19 Cost Model (First wave)

Given the regular updates to the NCCM’s structure and inputs and the ongoing changes to the country’s COVID-19 management policies and clinical guidelines, model results changed almost weekly for the duration of the first wave. Amongst the many updates, we report on a version from the end of May 2020, which used input from the first NCEM version to inform an additional allocation to COVID-19 related healthcare for the 2020/21 financial year.

The NCCM estimated that the budget required for the COVID-19 health response for financial year 2020/21 would be around 2.1 and 2.7 billion USD under the NCEM’s optimistic and pessimistic scenarios, respectively (Table 5). Scenarios differed in the cost of those budget items that were directly linked to the number of projected cases, particularly for inpatient care (ICU and non-ICU beds, ventilators and oxygen) and drugs at all levels of the healthcare system. In both scenarios, the largest contributor to total cost were the procurement and staffing of additional ICU beds (21% and 26%, resp., in the optimistic and pessimistic scenario), personal protective equipment (PPE) for healthcare workers at all levels (18% and 14%) and prefabricated facility infrastructure discussed at that time. This included additions to primary healthcare clinics for the management of non-severe patients (“fever clinics”) (11% and 9%) and 30-bed stand-alone COVID-19 wards as additions to hospitals (15% and 12%).

**Table 5:** The projected COVID-19 health budget for financial year 2020/21. Note that not all of these components were necessarily supported or included in the final allocations in the order of 1.5 billion USD.

Budget item	Description	Scenario with 20,000 additional ICU beds	
		Total cost [millions 2020 USD]	% of total cost
PPE	Personal protective equipment for healthcare workers at all levels	360	18%
Testing	PCR tests only; no new laboratory instruments or extra staff	145	7%
Central functions	Port Health and surveillance	23	1%
Intensive Care Unit beds	incl. additional beds required, linen and staff costs	426	21%
Ventilators	Additional ventilation equipment required for inpatient care	74	4%
Oxygen	Oxygen cylinders for inpatient care, excludes oxygen equipment	179	9%
Hospital beds	incl. additional beds required, linen and staff costs	9	0%
Drugs	at ICU, general wards and primary healthcare clinics	211	10%
PHC staff	for screening, testing, clinical assessment, post-test follow-up	32	2%
CHW supplies	1.1 thermometers per community healthcare worker	7	0.3%
Isolation facilities	Upgrading and repurposing of hotel and conference facilities to be used as facilities for isolation of mild cases	60	3%
Fever clinics	1000 units to be added to primary health centres and community health centres	218	11%
30-bed COVID-19 wards	Attached to existing hospital or to field hospital	299	15%
**TOTAL**		**2,043**	

Based on these results, about 1.5 billion USD was added under the COVID-19 Special Adjustment Budget in June 2020, funded through a combination of reprioritisation of funds from other departments and within the provincial health budgets and increased lending [7]. Most of this budget was used in expenditure on inpatient care and PPE, although over 12 months instead of the originally estimated 6 months, and allocated slightly differently. Few of the large infrastructure projects were implemented, except for the construction of three large field hospitals in the worst-hit provinces, in two cases through the repurposing of existing buildings, and with completion so delayed that very few patients were admitted. In total, fewer ICU beds were added due to severe constraints in the trained staff needed to staff them.

### Findings from resurgence modelling, forecasts and scenario modelling (Wave 2 onwards)

The resurgence monitoring dashboard provided continuous updates three times a week on the state and growth of the epidemic across provinces, districts and sub-districts from December 2020 onwards. The metrics on the dashboard classified all areas as being in a state of control, alert or response, which triggered a series of actions laid out in a Department of Health workplan [5]. The short-term forecasts provided two-week forecasts on the trajectory of cases and admissions at the provincial level from May 2021 onwards. These projections were placed within the context of the relationship between weekly admissions and hospital-based case fatality rates that was seen in prior waves, with one notable exception: At the beginning of the fourth wave (Omicron), we did not provide admissions forecasts due to uncertainty regarding the relationship between cases and admissions under this novel variant.

The scenario modelling for the third wave accounted for province-level data on seroprevalence and the dominance of new variants. The modelling estimated that, across most provinces and behavioural scenarios, the peak admissions of the third wave would be lower than that of the second wave. However, we saw that a slow, weak behavioural response would increase admissions for severe or critical COVID-19 cases across most age groups. Younger age groups were expected to have fewer admissions than in the second wave in most scenarios. Our analysis by province showed substantial variation in the size of the third wave between provinces, reflecting different age distributions, seroprevalence and prevalence of comorbidities. The third wave was highest in more urban provinces due to the higher concentration of working-age adults and people with co-morbidities. Across provinces, the time from an initial increase in transmission to the peak was estimated to be 2–3 months on average. A full report detailing scenario projections for each of the nine provinces is available in [45,46].

Lastly, our scenarios for the fourth wave projected that hospital admissions during the fourth wave would be less than during the third wave. This scenario considered a large range of assumptions regarding immune escape properties and transmissibility, a hypothetical new variant, loss of protection against severe infection and increase in contacts. The projected reduction in hospital admissions was in large part due to high population levels of combined immunity from previous infection and, to a lesser extent, vaccination (owing to low vaccine coverage at the time). This remained true in our updated sets of scenarios incorporating the higher transmissibility and immune evasion properties of Omicron compared to previous variants.

### Policy impact

While we are confident that our projections have provided important data to the evidence on which decisions and actions of the South African government were based, we are equally aware that they were merely one aspect that comes to bear on how these decisions were derived and which actions were implemented.—Other factors with a bearing on government decision making were clinical evidence, economic and social considerations, and aspects of political and practical feasibility. We can, therefore, claim no direct causal link between our data and any of the actions of the South African government, including decisions regarding what public health and social measures to implement at what stage. We do know, however, that staff within the national and provincial departments of health used our projections throughout the pandemic to plan for and reallocate required resources. Moreover, since December 2020our resurgence monitoring application was used to plan for local additional waves. Equally, SACMC members were part of the Ministerial Advisory Committee on COVID-19 and of a working group of the Ministerial Advisory Committee on COVID-19 Vaccines, and contributed to some of these advisories.

## Discussion

The process of providing policy-relevant research on the evolution and impact of an aggressive novel pathogen required a relevant, timely and responsive modelling approach; ongoing re-assessment of the availability of local and international data and evidence; and effective, transparent navigation of uncertainty. Partly based on above, this section gives an overview of the lessons we learned through our experience providing modelling and analytics support throughout the COVID-19 epidemic in South Africa, and expands these lessons to hold value for future pandemic preparedness efforts in South Africa and other low- or middle-income countries.

### The modelling approach needs to be relevant and responsive to evolving policy priorities

Over the course of an epidemic, the type of questions that are most important to decision makers changes. Modellers need to work closely with model users so that the outputs developed are relevant and timely, serving the dynamic needs of the user. Further, establishing strong relationships and embedding continuous engagement with stakeholders throughout the modelling process is fundamental to accelerating the use of modelling output into decision making. In South Africa, policy needs changed over time. At first, urgent long-term projections of the shape and peak of the first wave were required. Then, the need shifted to modelling the impact of behaviour to explain how the first wave had diverted from expectation. Subsequently, there was a need for real-time resurgence monitoring during the ensuing waves (each driven by a new variant). This new approach also needed to incorporate all previous learnings into third wave projections (alongside two-week forecasts of hospital-relevant data). Furthermore, just ahead of the fourth wave, scenarios were provided that included a potential novel variant with immune escape properties. Finally, we updated our fourth wave scenarios with the results of our own rapid-fire analysis of Omicron properties within days of the emergence of this variant. The approach discussed in this paper demonstrates the value of the adaptive nature of our policy-driven modelling work.

### Modellers need to be flexible in the tools they apply

At different stages of the epidemic, the varying availability of data and most pressing policy questions should inform the selection of an appropriate modelling or analytics approach. Short-term modelling can account for rapid and frequent changes, quickly incorporating new data and enabling a short lead time between updates and communication of findings. This is particularly relevant for important but rapidly changing inputs such as available hospital capacity [42–44]. Additionally, our initial projections had intentionally projected the number of beds *needed* without taking capacity and cost constraints into account, the actual capacity to accommodate severe and critical cases was much lower. As such, we added outputs (including budgets) based on actual bed *use*. The ability to project both bed need and bed use during the first wave, predict the extent of the health burden across provinces during the third and fourth waves, and monitor resurgence patterns and bed occupancy at the sub-district level, were possibly amongst our most useful contributions. However, even these outputs’ usefulness was limited by the non-fungibility of healthcare resources in many instances. The strongest constraint on inpatient bed availability was human resources, which could not be easily shifted and much less created over a short time span. Additionally, more than two thirds of deaths happened outside of hospitals, potentially pointing to COVID-19 patients being disheartened by reports of locally overwhelmed hospitals [47–49], being turned away by overburdened emergency rooms, becoming too sick too quickly to seek care in time, or dying in transit where emergency transport was scarce.

### Modellers need to make judgement calls about when to produce new projections- and if at all

We learned that it is in the hands of the modeller to judge if sufficient data are available and/or modelling can responsibly support the decisions to be made. Ahead of the second wave in South Africa in October 2020, model predictions of the shape and timing of the peak of this second wave were urgently requested. The SACMC decided to first assess the driving forces behind the resurgence, considering factors such as increased mobility, PHSM fatigue, lower seroprevalence or new variants. In the absence of additional information at the time (with the Beta variant only discovered in December 2020), the SACMC made the difficult decision not to produce model-based projections. Instead, we choose to develop a set of metrics that could detect and monitor the second wave. Further updates to the NCEM were only resumed in anticipation of the third wave and the vaccine roll-out programme, once more information was available. At the beginning of the fourth wave, our use of epidemiological tools such as reinfection analysis [50] allowed us to quickly produce bounding estimates for Omicron’s combined transmissibility and immune escape properties [51], which in turn provided reliable estimates of bed needs for this wave.

### Models should take local context into account

There are several challenges to modelling in low- and middle-income countries in general. Data systems and surveillance infrastructure are often underdeveloped. Constrained resources, especially during health emergencies, lead to overwhelmed hospital staff who are unable to feed data systems in real time. This can cause delays and errors in reporting. Heterogeneity in population characteristics and access to health infrastructure are vital elements in the disease ecosystem and essential inputs to disease models. Infectious disease models should account for the local context, such as data availability, health systems dynamics, demography, contact patterns, acceptability of interventions and cultural influences. In countries where data availability is scarce, it is important to understand what adaptations are needed and how models may perform under different data constraints, and to communicate the resulting uncertainty effectively. As such, the adaptation of plug-and-play models from resource-richer countries remains an inferior option, as these are usually ill-equipped to provide reliable, ongoing, real-time decision-making support tailored to local needs, data and epidemic specifics. One example of this is our mis-specification of mortality rates in v1 of our model which was a result of a misinterpretation of international data on case fatality rates, which were the only available data in March/April 2020 [42]. Incidentally, updating the model with locally derived mortality rates after the first South African wave resulted in very similar, albeit more correct, estimates of the number of overall deaths [44]. Another is our decision to not incorporate the impact of individual PHSM, despite clear and regular requests from policy makers to help with decisions regarding which individual restrictions would still be necessary. This decision was taken because, despite regular reviews, we did not find a robust enough dataset applicable to a low- or middle-income setting or South Africa specifically regarding the impact of these measures.

### Communicating uncertainty clearly and transparently is vital when reporting model findings

Managing expectations regarding the limitations of models, the quality of data and the assumptions used, improves the likelihood of model outputs being used responsibly and appropriately by decision makers. We used several widely accepted approaches for doing this, including using scenario and sensitivity analyses and clearly marked uncertainty ranges for input parameters and results. We found that uncertainty, while central to our understanding of our role as modellers, was not always useful to our audience of health planners. Especially where additional health resources had to be made available, users reverted to simply using our median estimates to inform budgets and order exact quantities. By the same token, we realised that communicating our methods and the sources of our assumptions to the general public was equally fundamental to building their trust in our results—though it was somewhat harder.

## Conclusion

In developing the NCEM, NCCM and a number of dashboards and additional outputs such as reports and briefing materials, the SACMC supported national and provincial government to plan several months ahead, expanding hospital facilities where needed, and procuring additional resources. As the country is continuing its path towards endemicity, the SACMC continues to serve the planning needs of the government, tracking the development of cases and admissions and developing models to further support the national vaccine rollout.

Disease modelling was a source of regularly updated scientific evidence for decision making in the South African epidemic. Much progress was made in developing models rapidly in an emergency setting. However, many challenges remain and need to be overcome to incorporate local context, needs of policymakers and sub-optimal data systems, and to build disease modelling capacity to better prepare for future health emergencies.

## Supporting information

S1 Appendix (PDF)
