# Supplementary material for: The role of modelling and analytics in South African COVID-19 planning and budgeting
Source: PLOS Glob Public Health. 2023 Jul 3;3(7):e0001063. doi: 10.1371/journal.pgph.0001063 (PMC10317222; doi:10.1371/journal.pgph.0001063)
Supplement: S1 Appendix — (PDF) [file pgph.0001063.s001.pdf]

Supplementary material
for

The Role of Modelling and Analytics in South African COVID-19 Planning and Budgeting

Gesine Meyer-Rath, Rachel A Hounsell, Juliet RC Pulliam, Lise Jamieson, Brooke E Nichols, Harry Moultrie, Sheetal P Silal

Table A: SACMC contributions over time (2020-2021)

Date	*Event*	*Description*
05/03/2020	1st COVID-19 case in SA	First case in South Africa reported
23/03/2020	SACMC formed	SACMC formed by the National Department of Health (NDOH). Core modelling team comprised of modellers from MASHA, SACEMA, HE^2RO and NICD
03/04/2020	1st NCCM results	First cost results shared with policy makers
14/04/2020	1st NCEM projections	First short- and long-term provincial- and national-level epi projections shared with policy makers
06/05/2020	NCEM public release (1,2)	First reports on short- and long-term epi projections made public
06/05/2020	NCCM budget projections	Data for COVID-19 health budget for 2020/21 shared with NDOH
23/05/2020	Special Adjustment Budget	COVID-19 health budget for 2020/21 announced
12/06/2020	NCEM public release (3)	Updates to short-term epi projections made public
03/07/2020	NCEM district projections	District-level long-term epi projections shared with policy makers
10/07/2020	NCEM Dashboard	Launch of National COVID-19 Epi Model Dashboard
21/07/2020	Scenarios (1)	Updates to long-term projections incorporating behavioural scenarios shared with policy makers
15/08/2020	Scenarios (2)	Updates to long-term projections incorporating behavioural heterogeneity shared with policy makers
06/09/2020	NCEM public release (4)	Report on long-term projections incorporating behavioural scenarios made public
17/09/2020	COVAX CEA	Cost-effectiveness analysis of accessing COVID-19 vaccines through COVAX
17/11/2020	SACMC Epidemic Explorer	Launch of SACMC Epidemic Explorer
21/11/2020	1st vaccination budget	First full vaccination budget estimates
15/01/2021	SACMC provincial reports (1)	Overview of second wave dynamics by province shared with provincial NDOH staff and advisors
18/01/2021	SACMC brief (1)	First epidemic update brief to MAC and policy makers
26/01/2021	NCCM 2021/22 budget	Data for COVID-19 health budget for 2021/22 shared with NDOH, incorporating potential additional waves
06/04/2021	3rd wave projections (1)	Report on 3rd wave projections considering hypothetical variant scenarios shared with policy makers
29/04/2021	NCEM public release (5)	Report on 3rd wave projections considering hypothetical variant scenarios made public
03/05/2021	SACMC memo (1)	Guide to interpretation of resurgence metrics shared with provincial- and national-level policy makers
04/05/2021	SACMC brief (2)	Epidemic update brief to MAC and policy makers

Table S1 cont'd

Date	*Event*	*Description*
14/05/2021	SACMC brief (3)	Epidemic update brief to MAC and policy makers
14/05/2021	SACMC memo (2)	Expectations for the timing of the third wave shared with MoH
26/05/2021	Short-term forecasting	Results of short-term forecasts shared with policy makers, and shortly thereafter launched on Epidemic Explorer
09/06/2021	SACMC brief (4)	Epidemic update brief to MAC and policy makers
06/07/2021	3rd wave projections (2)	Report on 3rd wave projections considering Delta variant shared with policy makers
08/07/2021	SACMC brief (5)	Epidemic update brief to MAC and policy makers
13/07/2021	NCEM public release (6)	Report on 3rd wave projections considering Delta variant made public
01/08/2021	Tekanelo inception	Begin of new project to jointly consider the epi and macro-economic impact of COVID-19
11/08/2021	SACMC brief (7)	Epidemic update brief to MAC and policy makers
04/09/2021	SACMC brief (8)	Epidemic update brief to MAC and policy makers
28/10/2021	NCEM 4th wave scenarios	Report on 4th wave projections considering hypothetical new variant scenarios
25/11/2021	Reinfection signal	Presentation on new signal in reinfections analysis suggesting emergence of a new immune escape variant
28/11/2021	SACMC brief (9)	Epidemic update brief to MAC and policy makers
03/12/2021	Omicron transmissibility & immune escape brief	Updated brief to IMT on bounding transmissibility and immune escape of the Omicron variant
13/12/2021	NCEM Omicron scenarios	Updated 4th wave projections considering emerging Omicron variant characteristics
15/12/2021	SACMC provincial reports (2)	Situation reports on trends in hospital admissions in all provinces shared with provincial- and national-level policy makers

National COVID-19 Epi Model (NCEM) adaptations from March 2020 to December 2021

The NCEM is a stochastic compartmental transmission model to estimate the total and reported incidence, admissions and deaths of COVID-19 in the nine provinces of South Africa. The outputs of the model may be used to inform resource requirements and predict where gaps could arise based on the available resources within the South African health system. The model follows a generalised Susceptible-Exposed-Infectious-Recovered (SEIR) structure accounting for disease severity (asymptomatic, mild, severe and critical cases) and the treatment pathway (outpatients, non-ICU and ICU beds). Figures S1-6 detail the structural development of the NCEM from March 2020 to December 2021 in response to requests for model outputs and the drivers of infection in South Africa.

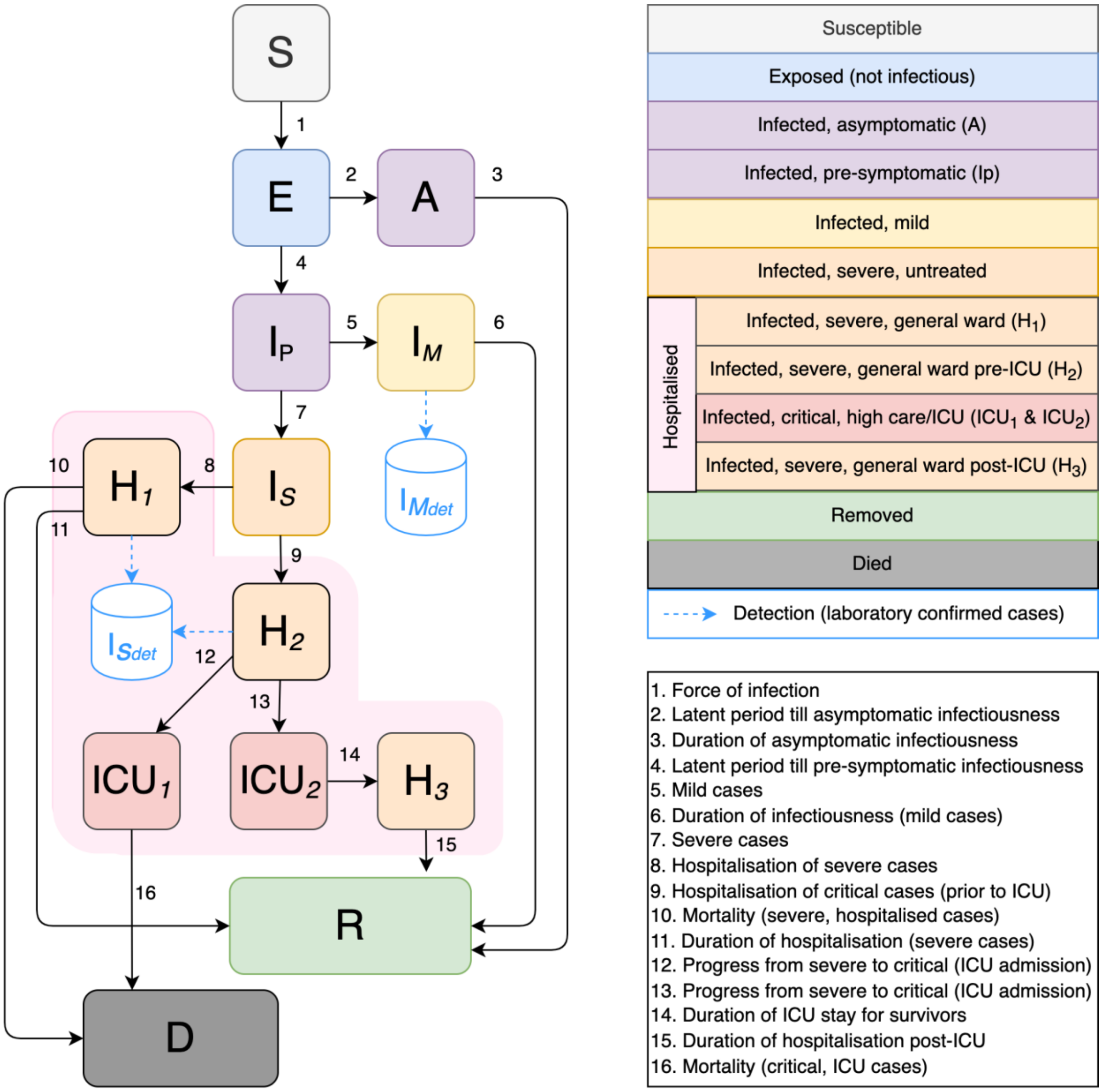

***Figure A: NCEM v1** Dynamic compartmental structure accounting for clinical profile of infection and hospital pathway*

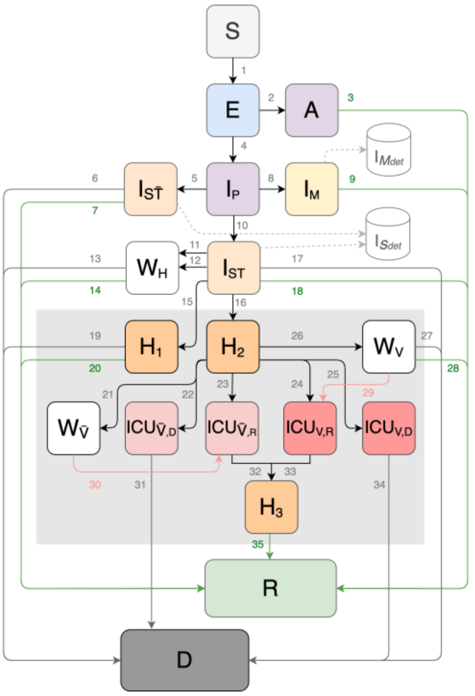

Model States

S		Susceptible
E		Exposed (not infectious)
A		Infected, asymptomatic
I_P		Infected, pre-symptomatic
I_M		Infected, mild
$I_{S\bar{T}}$		Infected, severe, untreated
I_{ST}		Infected, severe, seeking treatment
W_H		Infected, severe, waiting for hospital bed
Hospitalised	H_1	Infected, severe, general ward
	H_2	Infected, severe, general ward pre-ICU
	$W_{\bar{V}}$	Infected, critical, waiting for ICU, no ventilation
	$ICU_{\bar{V},D}$	Infected, critical, in ICU, not ventilated, non-survivor
	$ICU_{\bar{V},R}$	Infected, critical, in ICU, not ventilated, survivor
	W_V	Infected, critical, waiting for ICU & ventilation
	$ICU_{V,R}$	Infected, critical, in ICU, ventilated, survivor
	$ICU_{V,D}$	Infected, critical, in ICU, ventilated, non-survivor
	H_3	Infected, severe, general ward post-ICU
R		Removed (recovered)
D		Died
I_{Mdet}		Detection of mild cases (laboratory confirmed)
I_{Sdet}		Detection of severe cases (laboratory confirmed)

Model Flows

1. Force of infection
2. Latent period (until asymptomatic infectiousness)
3. Recovery: duration of asymptomatic infectiousness)
4. Latent period (until symptomatic infectiousness)
5. Development of severe symptoms, does not seek treatment
6. Death of severe, untreated case
7. Recovery: duration of severe case's infectiousness
8. Development of mild symptoms
9. Recovery: duration of mild case's infectiousness
10. Development of severe symptoms, seeks treatment
11. Severe case waiting for a hospital bed (if bed capacity reached)
12. Critical case waiting for a hospital bed (if bed capacity reached)
13. Death while waiting for hospital bed (excess mortality)
14. Recovery while waiting for hospital bed
15. Severe case admitted to hospital
16. Critical case admitted to hospital (pre-ICU progression)
17. Death of severe case while seeking treatment
18. Recovery of severe case while seeking treatment
19. Death of severe case in general hospital bed
20. Recovery of severe case in general hospital bed
21. Critical case in hospital, waiting for ICU admission (no ventilation)
22. Progression to ICU admission (no ventilation), non-survivor
23. Progression to ICU admission (no ventilation), survivor
24. Progression to ICU admission (with ventilation), survivor
25. Progression to ICU admission (with ventilation), non-survivor
26. Critical case in hospital, waiting for ICU admission (ventilation)
27. Death of critical case while awaiting ICU & ventilation
28. Recovery of critical case while awaiting ICU & ventilation
29. Waiting critical case needing ventilation admitted to ICU
30. Waiting critical case not needing ventilation admitted to ICU
31. Death of critical case from ICU (not ventilated)
32. Critical case discharged from ICU (non-ventilated) to general ward
33. Critical case discharged from ICU (ventilated) to general ward
34. Death of critical case from ICU (ventilated)
35. Recovery of critical case (discharged from hospital)

Figure B: NCEM v2-3 *Dynamic compartmental structure accounting for clinical profile of infection and hospital pathway. Additional features in v2 include limits on hospital capacity, <100% medical attendance for severe illness and ICU hospital pathway stratified by use of mechanical ventilation. NCEM v3 expands this structure through a spatially-explicit application to 52 districts in South Africa.*

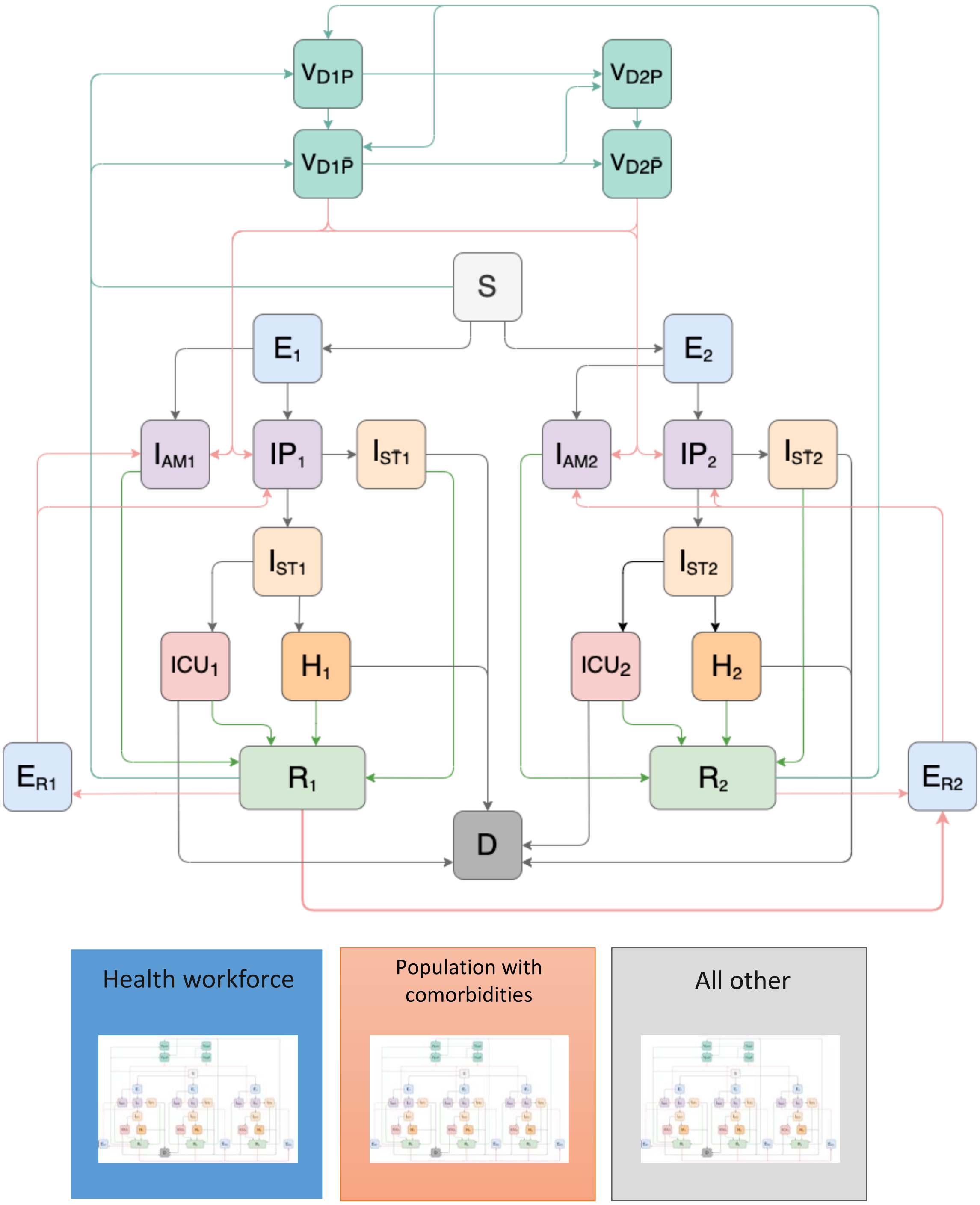

Figure C: NCEM v4 *Dynamic compartmental structure accounting for clinical profile of infection and hospital pathway. Additional features in v4 include a stratification by strain of infection to account for transmission of both Wild type SARS-CoV-2 and the Beta variant. Reinfection and cross-infection are also included. The model is additionally stratified by 7 age groups and three subpopulations. Vaccination is included to account for vaccine effectiveness against infection and severity for a generic vaccine.*

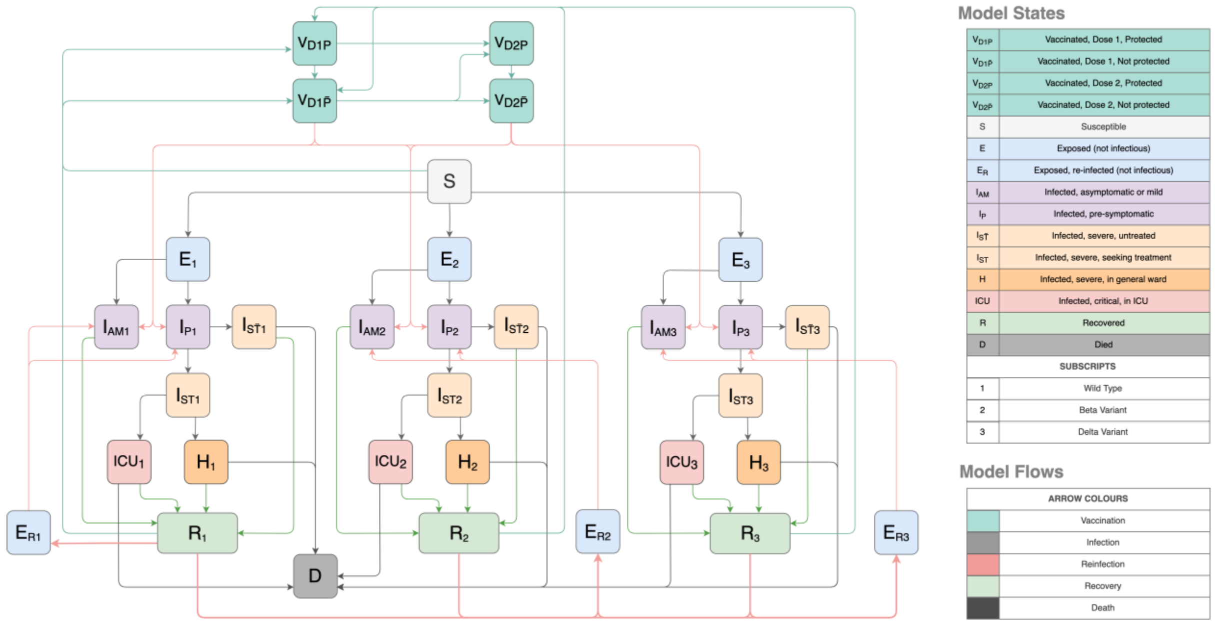

Figure D: NCEM v5 *Dynamic compartmental structure accounting for clinical profile of infection and hospital pathway. Additional features in v5 an additional stratification for transmission of the Delta variant.*

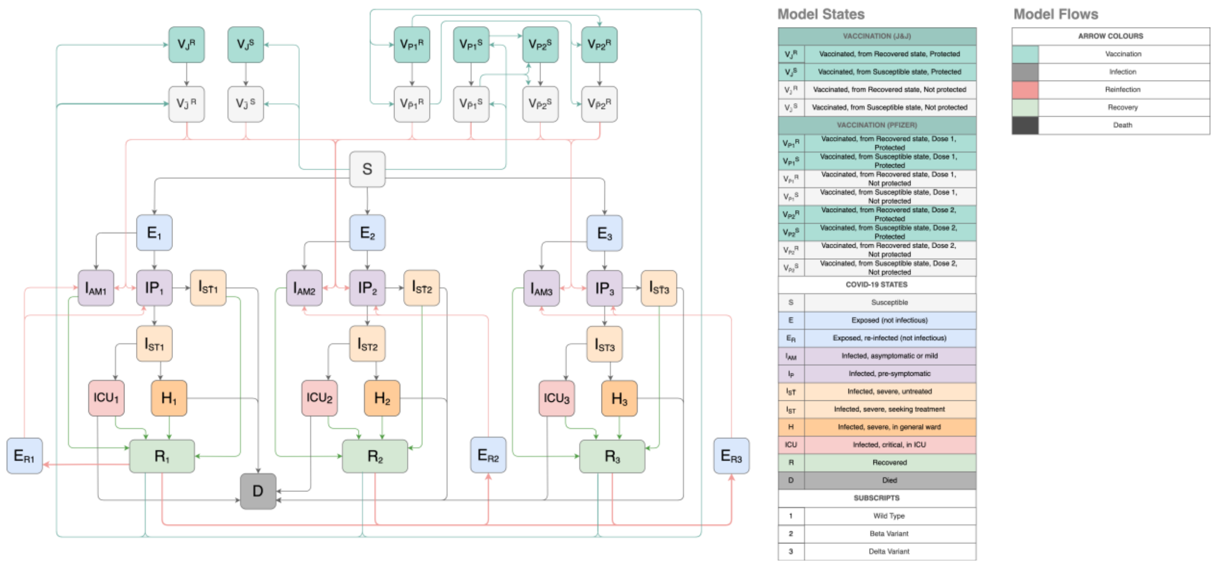

Figure E: NCEM v6 *Dynamic compartmental structure accounting for clinical profile of infection and hospital pathway. Additional features in v6 include vaccination pathways for specific vaccines (Corminarty and Janssen), accounting for differential vaccine efficacy based on previous infection*

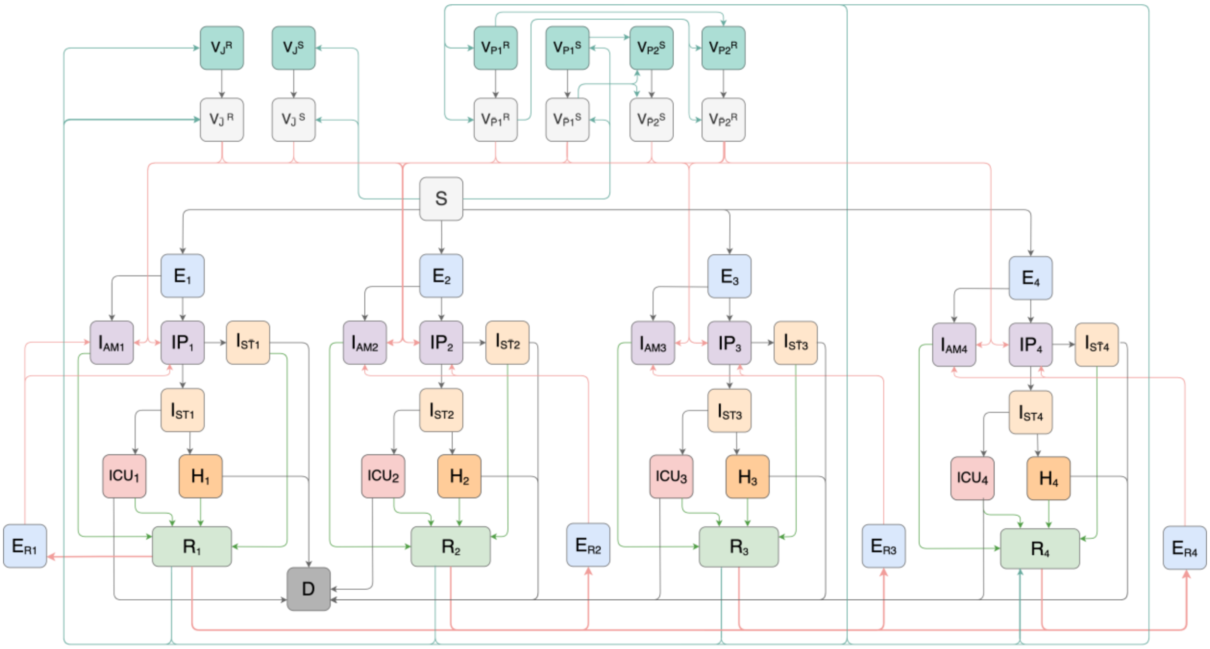

Transmission States

COVID-19 STATES	
S	Susceptible
E	Exposed (not infectious)
E_R	Exposed, re-infected (not infectious)
I_{AM}	Infected, asymptomatic or mild
I_P	Infected, pre-symptomatic
$I_{S\bar{T}}$	Infected, severe, untreated
I_{ST}	Infected, severe, seeking treatment
H	Infected, severe, in general ward
ICU	Infected, critical, in ICU
R	Recovered
D	Died

Vaccination States

VACCINATION (J&J)	
V_J^R	Vaccinated, from Recovered state, Protected
V_J^S	Vaccinated, from Susceptible state, Protected
$\bar{V}_J^R$	Vaccinated, from Recovered state, Not protected
$\bar{V}_J^S$	Vaccinated, from Susceptible state, Not protected
**VACCINATION (PFIZER)**	
V_{P1}^R	Vaccinated, from Recovered state, Dose 1, Protected
V_{P1}^S	Vaccinated, from Susceptible state, Dose 1, Protected
$\bar{V}_{P1}^R$	Vaccinated, from Recovered state, Dose 1, Not protected
$\bar{V}_{P1}^S$	Vaccinated, from Susceptible state, Dose 1, Not protected
V_{P2}^R	Vaccinated, from Recovered state, Dose 2, Protected
V_{P2}^S	Vaccinated, from Susceptible state, Dose 2, Protected
$\bar{V}_{P2}^R$	Vaccinated, from Recovered state, Dose 2, Not protected
$\bar{V}_{P2}^S$	Vaccinated, from Susceptible state, Dose 2, Not protected

Variants

SUBSCRIPTS	
1	Wild Type
2	Beta Variant
3	Delta Variant
4	Omicron Variant

Transitions

ARROW COLOURS	
(teal)	Vaccination
(grey)	Infection
(red)	Reinfection
(light green)	Recovery
(dark grey)	Death

Figure F: NCEM v7 *Dynamic compartmental structure accounting for clinical profile of infection and hospital pathway. Additional features in v7 include an additional stratification for the Omicron variant.*
